# Vitamin D deficiency and uterine leiomyoma in unexplained infertility

**DOI:** 10.17305/bb.2025.12952

**Published:** 2025-09-19

**Authors:** Yüksel Onaran, Esra Goktas, Beyza Altın Öztürk, Serkan Kahyaoglu, Hatice Akkaya

**Affiliations:** 1 Department of Obstetrics and Gynecology, Bilkent City Hospital, University of Health Sciences, Ankara, Türkiye; 2 Department of Obstetrics and Gynecology, Aksaray University Training and Research Hospital, Aksaray, Türkiye; 3 Department of Obstetrics and Gynecology, Bilkent City Hospital, Ankara, Türkiye

**Keywords:** Vitamin D, leiomyomas, infertility

## Abstract

Uterine leiomyomas are the most common benign tumors of the female genital tract, and alongside hormonal and genetic factors, emerging evidence implicates vitamin D deficiency in their pathogenesis. We investigated the association between serum 25-hydroxyvitamin D [25(OH)D] and the presence of uterine leiomyomas in women with unexplained infertility. In this retrospective case–control study, 148 women aged 18–45 years presenting to the Infertility Clinic of Ankara Bilkent City Hospital between July 2019 and February 2024 were included: 74 had imaging-confirmed leiomyomas (non-submucosal; FIGO types 4–6) and 74 infertile controls had no leiomyomas. Serum 25(OH)D was measured and demographic/clinical data were analyzed with appropriate parametric and non-parametric tests; correlations used Spearman’s rho, and an ANCOVA adjusted for body mass index (BMI) and season assessed group differences. Groups were comparable in age and BMI (e.g., age 35.08 ± 5.79 vs 33.30 ± 5.57 years; *P* ═ 0.062). Mean serum 25(OH)D was significantly lower in women with leiomyomas than in controls (41.4 ± 23.7 vs 62.0 ± 34.2 nmol/L; *P* < 0.001), and this difference remained significant after adjustment for BMI and season (ANCOVA F = 10.7, *P* ═ 0.001). Vitamin D levels did not differ by leiomyoma number (single vs multiple: 44.1 ± 21.6 vs 38.5 ± 25.83 nmol/L; *P* ═ 0.32) or location (intramural vs subserosal: 40.7 ± 24.9 vs 43.1 ± 21.1 nmol/L; *P* ═ 0.69), and were not correlated with leiomyoma size (Spearman *r* ═ −0.04; *P* ═ 0.70). Among women with unexplained infertility, uterine leiomyomas are thus associated with significantly lower serum 25(OH)D levels, independent of BMI and season, whereas vitamin D status is unrelated to leiomyoma number, size, or location. These findings support a potential role of vitamin D deficiency in leiomyoma pathogenesis and underscore the need for larger, multicenter prospective studies to clarify causality and clinical implications.

## Introduction

Uterine leiomyomas are the most common tumors of the female genital tract [1]. These benign monoclonal tumors arise from the smooth muscle cells and fibroblasts of the myometrium [2]. Their growth and development are influenced by progesterone, estrogen, and associated growth factors and proteins. Although the exact etiology of uterine leiomyomas remains unclear, ongoing research is investigating their molecular biology, hormonal influences, and genetic factors [3]. A systematic review by Stewart et al. [4] identified several factors that increase the incidence of leiomyomas, including high body mass index (BMI), Black ethnicity, family history, nulliparity, and reproductive age. In addition to these established risk factors, some studies suggest that low vitamin D levels may contribute to the formation of leiomyomas [5]. Research on the impact of vitamin D deficiency on leiomyomas has particularly focused on African–American women [6], owing to their higher melanin levels, which increase susceptibility to vitamin D deficiency [7]. Furthermore, decreased vitamin D levels have been associated with reduced expression of vitamin D receptors (VDRs) in adjacent myometrial tissue [8].

Uterine leiomyomas demonstrate variability in location, size, number, and symptomatology [4]. While a significant proportion of leiomyomas are asymptomatic, approximately one-quarter can lead to a range of severe and chronic symptoms [4]. The most prevalent symptom is abnormal uterine bleeding, which may result in secondary iron deficiency anemia. Other potential symptoms include pelvic pain, bloating, constipation, and obstetric complications [9].

Clinically symptomatic leiomyomas are typically managed through surgical interventions [10]. Surgical options encompass hysterectomy, myomectomy, and hysteroscopic resections [11]. Leiomyomas are a common indication for hysterectomy [12]. The primary objective of optimal treatment is to preserve fertility while minimizing blood loss and tumor burden [13]. Emerging evidence indicates that progesterone plays a crucial role in leiomyoma development and stimulates their growth to larger sizes [14]. This understanding has facilitated the development of ulipristal acetate (UPA), a selective progesterone receptor modulator (SPRM), as a significant pharmacological treatment for leiomyomas [15]. Due to their efficacy, UPA agents were initially employed as first-line therapy to prepare leiomyomas for surgery. However, the use of UPA has recently been curtailed due to concerns regarding hepatotoxicity. Additionally, UPA is an expensive treatment, and ongoing research is investigating the risk of liver failure with long-term use [16, 17].

Given the diverse origins of leiomyomas, an effective prophylactic method has yet to be established [18]. Although numerous studies have aimed to develop prophylactic strategies, a definitive method remains elusive [18]. Therefore, vitamin D prophylaxis, due to its significant role in the biological development of leiomyomas, may present a viable solution [19].

Vitamin D encompasses a group of fat-soluble steroid compounds that exert potent effects within the human body, with VDRs present in various organs, including the myometrium and leiomyomas [20]. The primary sources of vitamin D for humans include diet, dietary supplements, and sunlight. Vitamin D is synthesized in the skin from 7-dehydrocholesterol. The liver subsequently converts this molecule to 25-hydroxyvitamin D [25(OH)D], which is then converted by the kidneys to 1,25-dihydroxyvitamin D [1,25(OH)D]. Optimal serum vitamin D levels are defined as a 25(OH)D concentration of 50 nmol/L (20 ng/mL) [21]. Vitamin D is involved in regulating the cell cycle and cell differentiation and possesses anti-angiogenic properties [22]. Deficiency in vitamin D is emerging as a significant risk factor for the development of leiomyomas [23]. Various hypotheses have been proposed regarding the use of vitamin D in the prophylaxis and treatment of leiomyomas [24], but clinical studies in this area remain insufficient.

Abnormal serum levels of vitamin D have been observed in various gynecological and obstetric conditions, including polycystic ovary syndrome (PCOS), infertility, and preterm birth [25]. Unexplained infertility is defined as the inability to achieve pregnancy after 12 months of regular unprotected intercourse, despite normal results in standard infertility evaluations, including ovulation assessment, tubal patency, and semen analysis. Although routine investigations may not identify clear abnormalities, factors such as oocyte and sperm quality issues, fertilization failure, endometrial receptivity defects, immunological factors, genetic and epigenetic abnormalities, and subtle hormonal imbalances may contribute to unexplained infertility [26]. Currently, low serum vitamin D levels are considered a potential risk factor for the development of leiomyomas [19]. The notion that vitamin D plays a role in the pathogenesis of leiomyomas has gained increasing attention in recent years [27]. Recent studies have demonstrated a negative correlation between serum vitamin D levels and the presence of leiomyomas [6]. Low serum vitamin D levels in women have been associated with leiomyomas, regardless of ethnic origin [6]. Observations indicate that patients with adequate serum vitamin D levels are less likely to develop leiomyomas compared to those with deficient levels. Furthermore, sunlight exposure has been shown to decrease the risk of leiomyoma formation [6]. A correlation study found that patients with symptomatic leiomyomas exhibited lower vitamin D levels [7]. A study conducted in Türkiye similarly revealed lower serum vitamin D levels in patients with leiomyomas, although no correlation was identified between low vitamin D levels and the volume, location, or number of leiomyomas [28]. The first study to elucidate the effect of vitamin D on leiomyoma growth was published by Blauer et al. [20]. This research demonstrated a relationship between 1,25(OH)D levels and the growth of leiomyoma cells [20]. Inhibition of growth was correlated with vitamin D concentrations; increased vitamin D levels were associated with reduced leiomyoma growth [20]. Subsequently, another study by Sharan et al. [29] observed that 1,25(OH)D inhibited the proliferation of leiomyoma cells *in vitro*.

A study conducted by Al-Hendy et al. found that 1,25(OH)D demonstrates significant antiprogestogenic and antiestrogenic properties [29]. The research revealed an inverse correlation between the expression of estrogen and progesterone receptors and VDR levels. Additionally, the study indicated that active vitamin D treatment markedly reduced the levels of progesterone and estrogen receptors [29]. Furthermore, investigations by Halder et al. have shown a substantial decrease in leiomyoma growth associated with vitamin D [30, 31]. The authors concluded that 1,25(OH)D downregulates transforming growth factor beta 3 (TGF-beta3)-related gene expression, thereby slowing the growth of uterine leiomyomas and inhibiting the proliferation of leiomyoma cells [30, 31].

## Materials and methods

This retrospective case–control study examined patients who sought treatment for infertility at the Infertility Clinic of Ankara Bilkent City Hospital from July 2019 to February 2024. Participants included those with a confirmed diagnosis of leiomyoma via imaging techniques, specifically transvaginal ultrasonography using the GE Voluson E10, and an absence of tubal pathologies as confirmed by hysterosalpingography (HSG). All included leiomyomas were classified as types 4, 5, or 6, with no instances of submucosal fibroids (FIGO types 0, 1, 2, or 3). Each patient underwent office hysteroscopy as part of their infertility evaluation. Semen analyses were conducted, with participants exhibiting normal findings; those with abnormal results were excluded. Additionally, women diagnosed with hypogonadotropic hypogonadism, pituitary insufficiency, hyperprolactinemia, PCOS, or premature ovarian insufficiency (POI) characterized by low ovarian reserve were also excluded. The control group consisted of infertile individuals without leiomyomas and no other uterine or tubal pathologies, all of whom had documented vitamin D levels in the hospital’s patient record system.

**Figure 1.**
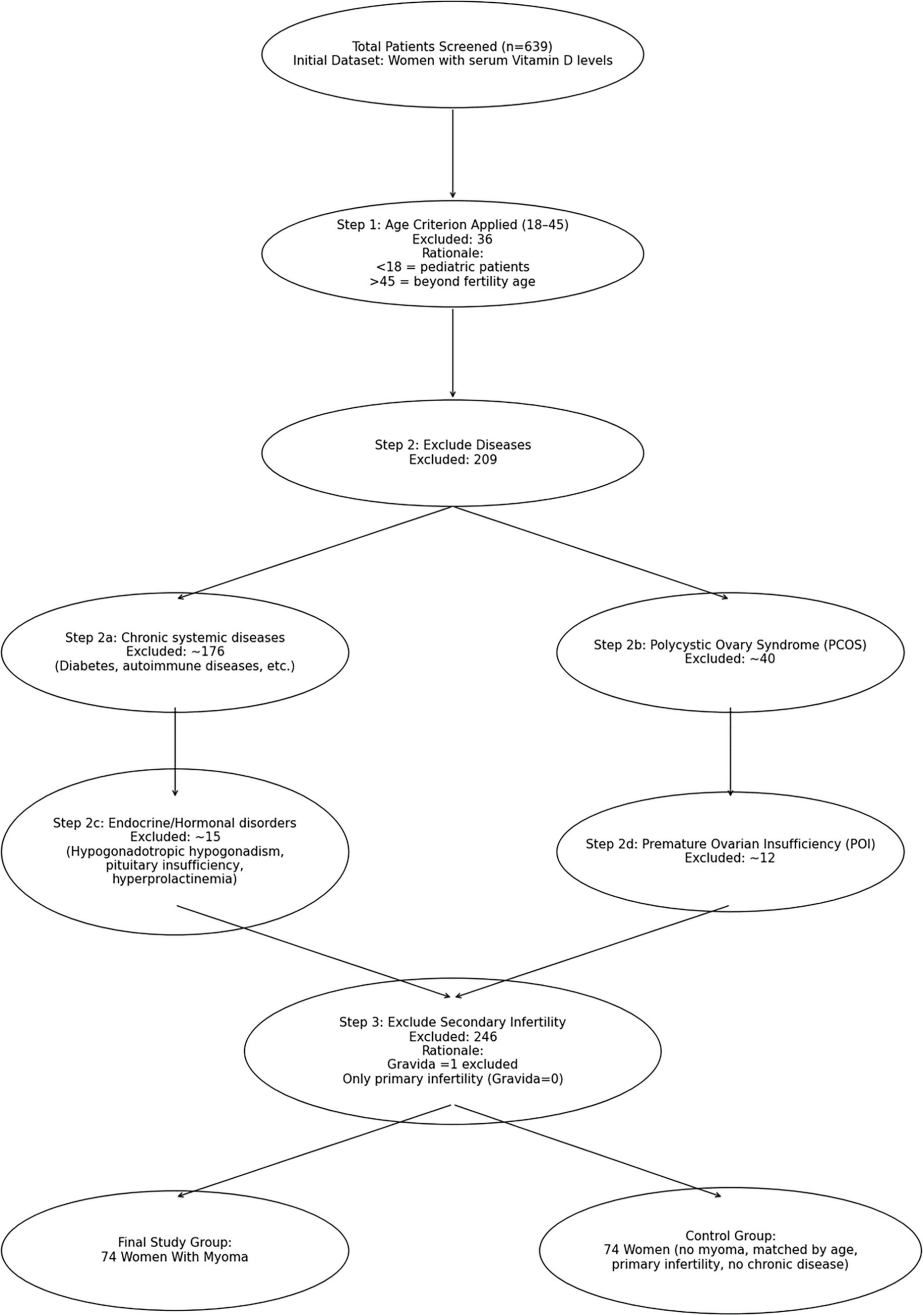
**Flow diagram of patient selection**.

A total of 148 individuals experiencing unexplained infertility were included in the study. Two groups were formed: 74 individuals with unexplained infertility and leiomyomas, and 74 individuals with unexplained infertility without leiomyomas. The control group was selected through sequential admissions at the polyclinic until 74 patients without leiomyomas were recruited for comparison (Figure 1).

Participants aged 18–45 years, diagnosed with primary infertility and confirmed to have leiomyoma via any imaging modality, were eligible for inclusion. Those taking vitamin D supplements and/or hormonal therapies, including oral contraceptives, as well as individuals with chronic systemic diseases, were excluded from the study.

Biochemical analyses were performed at the Central Biochemistry Laboratory of Ankara Bilkent City Hospital. Venous blood samples were collected from both the patient and control groups using gel serum tubes. All samples were obtained under standardized conditions to ensure consistency and minimize pre-analytical variability. Blood samples were drawn in the morning at the certified blood collection center of Ankara Bilkent City Hospital, with all participants instructed to fast overnight for at least 8–12 h prior to sampling. This protocol was uniformly applied across both groups to eliminate potential confounding factors related to circadian variation or postprandial biochemical fluctuations, particularly concerning vitamin D and metabolic parameters. After collection, samples were allowed to clot for 20 min before being centrifuged at 1300xg for 10 min. Total 25(OH)D vitamin levels were quantified using an immunoassay method on an Atellica IM analyzer (Siemens Healthineers, Mannheim, Germany).

Statistical analyses were conducted using SPSS for Windows version 21.0 (SPSS Inc., IL, USA). A 95% confidence interval was established, with *P* < 0.05 considered statistically significant. The assumption of normal distribution was assessed using the Shapiro–Wilk test. Data are presented as means ± SD for continuous variables. To evaluate differences across groups, the independent sample *t*-test and Mann–Whitney *U* test were employed. Correlations among parameters were determined using the Spearman correlation test. The statistical program available on the University of British Columbia’s statistical department website was utilized to compute the sample size and statistical power for the study (http://www.stat.ubc.ca/∼rollin/stats/ssize/n2.html).

According to this calculation, including 74 patients in each study group was deemed sufficient to achieve 80% statistical power at a significance level of *P* < 0.05.

### Ethical statement

The study was conducted in accordance with the Declaration of Helsinki and received approval from the Institutional Review Board (Ethics Committee) of Ankara Bilkent City Hospital (TABED 2-24-85, March 20, 2024).

**Figure 2.**
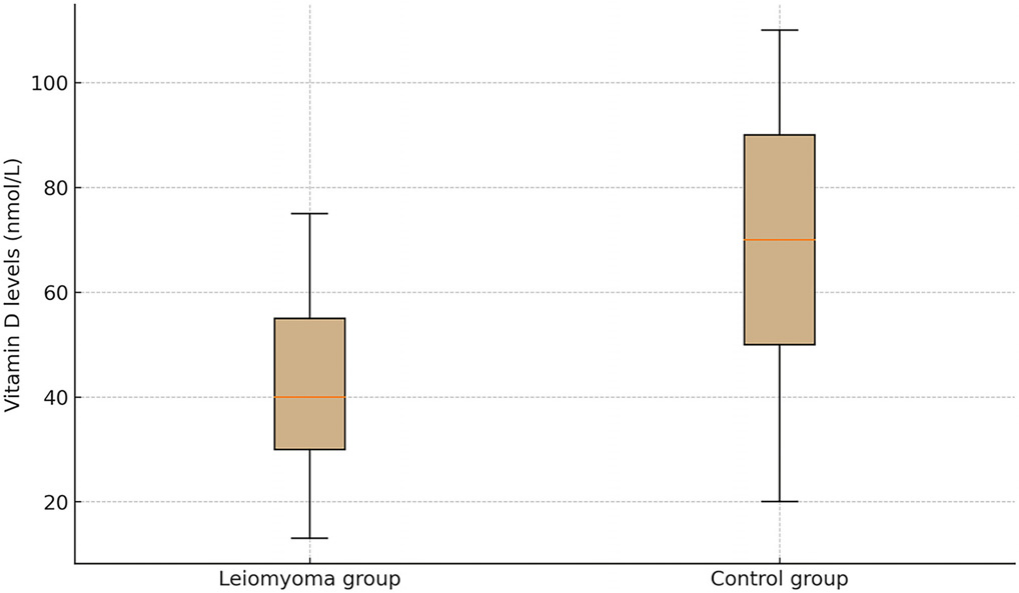
**Distribution of serum 25-hydroxyvitamin D [25(OH)D] levels in the leiomyoma and control groups.** The box plot depicts the median, interquartile range (IQR), and overall range of 25(OH)D concentrations (nmol/L) for each group. Mean ± SD values were 41.4 ± 23.7 nmol/L vs 62.0 ± 34.2 nmol/L, respectively; the between-group difference was statistically significant (*P* < 0.001).

## Results

A total of 148 patients with unexplained infertility were included in this study. Participants were divided into two groups: one consisting of 74 patients with unexplained infertility and leiomyomas, and the other comprising 74 patients with unexplained infertility without leiomyomas. The control group was selected through sequential admissions at the clinic until the number of patients without leiomyomas reached 74, matching the number in the study group.

The age range for the leiomyoma group was 22–42 years, while the age range for the control group was 23–38 years. Both groups included patients who were primarily nulligravid and experiencing infertility for the first time. The mean age of the study group was 35.08 ± 5.79 years, and the mean age of the control group was 33.3 ± 5.57 years, indicating no significant age difference between the two groups (*P* ═ 0.062) (see Table 1).

**Table 1:** Demographic data of patients

**Variables**	**Infertility with leiomyoma (*n* ═ 74)**	**Infertility without leiomyoma (*n* ═ 74)**	*P* value*
Age (year)	35.08±5.79	33.3±5.57	0.062
Weight (kg)	70.5±13	72.6±12.3	0.326
Height (cm)	164±6.1	164±6.0	0.77
BMI (kg/m^2^)	25.93±4.56	26.75±4.57	0.363
25(OH) Vit D (nmol/L)	41.4±23.7	62.0±34.2	<0.001
Adjusted 25(OH) Vit D level (nmol/L)	41.9±3.33	61.4±3.32	0.000

Note: Values are expressed as mean ± standard deviation. *Comparison between groups was performed using the Mann–Whitney *U* test. Abbreviations: BMI: Body mass index; 25(OH) Vit D: 25-hydroxyvitamin D.

No notable differences in sociodemographic characteristics were observed between the groups. The mean serum 25(OH)D levels were significantly different, with the study group averaging 41.4 ± 23.7 nmol/L and the control group averaging 62.0 ± 34.2 nmol/L (*P* < 0.001) (see Figure 2).

In the leiomyoma cohort, 38 cases (51.4%) presented with solitary leiomyomas, while 36 cases (48.6%) exhibited multiple leiomyomas. A comparative analysis of vitamin D levels between these groups revealed no significant difference; the solitary leiomyoma group had an average vitamin D level of 44.1 ± 21.6 nmol/L, compared to 38.5 ± 25.83 nmol/L in the multiple leiomyoma group (*P* ═ 0.32). Regarding the anatomical location of the leiomyomas, the majority were classified as intramural (70.3%, 52 cases) or subserosal (29.7%, 22 cases) with an intramural component. An analysis of vitamin D levels in relation to the location of the leiomyomas also showed no significant difference; the intramural group had an average vitamin D level of 40.7 ± 24.9 nmol/L, while the subserosal group had 43.1 ± 21.1 nmol/L (*P* ═ 0.69).

The correlation between leiomyoma size and vitamin D levels was assessed using the Spearman correlation test (*r* ═ −0.04, *P* ═ 0.7), indicating no statistically significant association. Additionally, no significant relationships were found between vitamin D levels and other variables, including the number, location, or size of leiomyomas (*P* > 0.05). The variance inflation factor (VIF) values ranged from 1.00 to 1.25, suggesting no significant multicollinearity among the predictors. The group difference in vitamin D levels remained significant after adjusting for potential confounders, such as BMI and seasonality.

An ANCOVA was performed to compare serum vitamin D levels between women with and without leiomyomas, adjusting for BMI and season. The analysis revealed that the difference in vitamin D levels between the two groups remained statistically significant after accounting for these covariates (ANCOVA, *F* ═ 10.7, *P* ═ 0.001). Adjusted vitamin D levels are presented in Table 1. The results of the multivariable regression analysis for determinants of vitamin D levels are shown in Table 2.

The reference intervals for vitamin D status utilized by the Bilkent City Hospital laboratory are defined as follows: deficiency (<50 nmol/L), insufficiency (50–75 nmol/L), and normal (75–250 nmol/L) (Table 3). The median serum vitamin D levels in the leiomyoma group were lower than those in the control group, aligning with a higher prevalence of deficiency and insufficiency.

**Table 2:** Multivariable regression analysis of factors associated with vitamin D levels

**Predictor**	**Std. Error**	**Beta (standardized coefficient)**	***t***	**Sig. (*P* value)**	**95% confidence interval (lower, upper)**
Age	0.471	−0.191	−1.66	0.102	[−1.721, 0.158]
BMI	0.587	−0.314	−27.75	0.007	[−2.799, -0.457]
Leiomyoma count (single/multiple)	5.850	−0.161	−1.3	0.198	[−19.2, 4.66]
Leiomyoma size	0.11	−0.015	−0.127	0.89	[−0.236, 0.208]
Leiomyoma site	6.16	−0.006	−0.047	0.96	[−12.58, 12.03]
Season effect	2.123	−0.197	−1.28	0.201	[−6.9, 1.469]

Abbreviation: BMI: Body mass index.

**Table 3:** Vitamin D level categorization based on provided reference levels

**Category**	**nmol/L**	**ng/mL**
Deficient	0–50	0–20
Insufficient	50–75	20–30
Normal	75–250	30–100

Note: The reference intervals of vitamin D status used in the laboratory of Bilkent City Hospital are as follows: Deficiency (<50 nmol/L), insufficiency (50–75 nmol/L), and normal (75–250 nmol/L).

## Discussion

VDRs are widely distributed throughout the human body, extending beyond organs primarily associated with calcium metabolism to encompass a diverse array of tissues and organs. Numerous studies have demonstrated that vitamin D plays a critical role in various biological processes within the body [32]. Didriksen et al. [33] suggested that genetic polymorphisms in vitamin D-related enzymes can significantly affect serum 25(OH)D levels. Lower levels of vitamin D have been proposed as a risk factor for the formation and growth of leiomyomas [6]. A study by Halder et al. reported that leiomyomas exhibit reduced VDR levels compared to normal myometrial tissue [19]. An inverse relationship has been observed between VDR levels and the upregulation of estrogen and progesterone receptors in leiomyomas, indicating that vitamin D may act as an antagonist to sex steroid hormones in this tissue [30, 34].

Our study revealed a statistically significant difference in serum 25(OH)D levels between patients with leiomyomas and a control group. However, when comparing cases with solitary vs multiple leiomyomas, no significant difference in serum 25(OH)D levels was found. This suggests that serum 25(OH)D levels are unrelated to the location, size, or number of leiomyomas. A 2018 study conducted in Türkiye reported findings consistent with ours; additionally, we demonstrated an association between vitamin D deficiency and infertility in women with leiomyomas [28]. A notable strength of our study is the screening of the patient group for leiomyomas by an experienced radiologist. Limitations include the unknown onset of the disease and the inability to assess vitamin D levels before and after the development of leiomyomas.

In summary, our study concludes that patients with leiomyomas have lower vitamin D levels, suggesting that vitamin D deficiency may contribute to the development of leiomyomas. To confirm these findings, further large-scale, multi-center studies with larger patient populations are warranted. Additionally, future research should investigate the therapeutic role of vitamin D supplementation in patients with uterine leiomyomas.

## Conclusion

This study demonstrates that serum 25(OH) vitamin D levels are significantly lower in patients with leiomyomas compared to those without, among individuals assessed for primary or secondary infertility. However, no significant association was identified between vitamin D levels and the location, number, or size of the leiomyomas. These findings suggest that vitamin D deficiency may serve as a risk factor in the development of leiomyomas. Given the limitations of this study, further large-scale, multicenter, and prospective research is necessary to clarify this relationship. In conclusion, while our findings indicate a potential link between vitamin D status and leiomyomas, additional studies are essential to elucidate the role of vitamin D in the reproductive system. If the connection between vitamin D deficiency and leiomyoma pathogenesis is confirmed, monitoring and correcting vitamin D levels could provide a promising strategy for leiomyoma management.
